# SOCIAL RELATIONSHIPS AND COGNITIVE IMPAIRMENT IN LATER LIFE

**DOI:** 10.1093/geroni/igac059.2635

**Published:** 2022-12-20

**Authors:** Patricia Thomas, Madison Sauerteig-Rolston, Shawn Bauldry, Samuel Nemeth, Kenneth Ferraro

**Affiliations:** Purdue University--West Lafayette, West Lafayette, Indiana, United States; Purdue University, West Lafayette, Indiana, United States; Purdue University, West Lafayette, Indiana, United States; Purdue University, West Lafayette, Indiana, United States; Purdue University, West Lafayette, Indiana, United States

## Abstract

With population aging and the growing population at-risk of cognitive impairment, Alzheimer’s disease, and related dementias, there is a need for a better understanding of how social factors may affect cognitive impairment. Drawing from stress process theory and social integration theory, we examine the impact of positive and negative dimensions of relationship quality with friends and family on cognitive impairment in later life. We analyze Cox proportional hazards models using nationally representative panel data from the Health and Retirement Study (2006-2016, N=10,626) to examine how the quality of family and friend relationships in adulthood may influence risk of cognitive impairment. Strain with family members and with friends was significantly related to higher risk of cognitive impairment (Hazard Ratio: 1.15 [95% CI: 1.08, 1.21] and 1.20 [95% CI: 1.13, 1.28], respectively). Support from family members was beneficial, related to lower risk of cognitive impairment (HR: 0.93, CI: .89, .99); however, support from friends was related to higher risk of cognitive impairment (HR: 1.10, CI: 1.05, 1.14). Findings reveal the implications of social support and strain in different types of relationships for cognitive health.

